# Quantifying combined effects of colistin and ciprofloxacin against *Escherichia coli* in an in silico pharmacokinetic-pharmacodynamic model

**DOI:** 10.1038/s41598-024-61518-0

**Published:** 2024-05-22

**Authors:** Chenyan Zhao, Anders N. Kristoffersson, David D. Khan, Pernilla Lagerbäck, Ulrika Lustig, Sha Cao, Charlotte Annerstedt, Otto Cars, Dan I. Andersson, Diarmaid Hughes, Elisabet I. Nielsen, Lena E. Friberg

**Affiliations:** 1 Department of Pharmacy, Uppsala University, Uppsala, Sweden; 2 Department of Medical Sciences, Uppsala University, Uppsala, Sweden; 3 Department of Medical Biochemistry and Microbiology, Uppsala University, Uppsala, Sweden

**Keywords:** Pharmacodynamics, Pharmacokinetics, Bacterial infection

## Abstract

Co-administering a low dose of colistin (CST) with ciprofloxacin (CIP) may improve the antibacterial effect against resistant *Escherichia coli*, offering an acceptable benefit-risk balance. This study aimed to quantify the interaction between ciprofloxacin and colistin in an in silico pharmacokinetic-pharmacodynamic model from in vitro static time-kill experiments (using strains with minimum inhibitory concentrations, MIC_CIP_ 0.023–1 mg/L and MIC_CST_ 0.5–0.75 mg/L). It was also sought to demonstrate an approach of simulating concentrations at the site of infection with population pharmacokinetic and whole-body physiologically based pharmacokinetic models to explore the clinical value of the combination when facing more resistant strains (using extrapolated strains with lower susceptibility). The combined effect in the final model was described as the sum of individual drug effects with a change in drug potency: for ciprofloxacin, concentration at half maximum killing rate (EC_50_) in combination was 160% of the EC_50_ in monodrug experiments, while for colistin, the change in EC_50_ was strain-dependent from 54.1% to 119%. The benefit of co-administrating a lower-than-commonly-administrated colistin dose with ciprofloxacin in terms of drug effect in comparison to either monotherapy was predicted in simulated bloodstream infections and pyelonephritis. The study illustrates the value of pharmacokinetic-pharmacodynamic modelling and simulation in streamlining rational development of antibiotic combinations.

## Introduction

Ciprofloxacin (CIP) is a fluoroquinolone antibiotic used to treat bacterial infections including urinary tract infections (UTIs). UTIs are caused by a broad spectrum of microorganisms such as *Escherichia coli*. However, CIP resistance has increased rapidly during the last decades^1,2^. Combination therapy holds the potential to improve the antibacterial effect in such cases. Colistin (CST), an old antibiotic mainly used as last resort drug against multidrug resistant Gram-negative bacteria, acts as a membrane disruptor by binding to surface molecules such as lipopolysaccharides and thus causing disintegration of bacterial membrane^3^, is expected to enhance the antibacterial effect of the co-administered drug by increasing membrane permeability and reducing efflux of the permeated antibiotics. Given the concern that CST has high incidences of nephrotoxicity at therapeutic doses^4,5^, a low CST dose would be preferred and may result in an acceptable benefit-risk balance when used in combination.

The combination of CIP and CST has been found to be effective against *Pseudomonas aeruginosa *in vitro against multidrug resistant and persistent pathogens^6–11^, and shown to eradicate early *P. aeruginosa* infection in cystic fibrosis patients^12^. Recent publications reported a synergistic effect in vitro against *Acinetobacter baumannii*^13^ and *Klebsiella pneumoniae*^14^. However, the combination effect against *E. coli* is unclear. A potential prophylactic effect of the combination against Gram-negative sepsis development, including *E. coli*, in neutropenic patients has however been reported^15^.

Pharmacokinetic-pharmacodynamic (PKPD) modelling uses mathematical and statistical models to quantitatively describe antibiotic dose-concentration-effect relationships based on experimental data^16^. A well-characterized relationship is essential for understanding the time-course of bacterial killing and resistance development, especially under combination therapy^17^ where the magnitude of the interaction depends not only on the fluctuating drug concentrations, but also on the dynamically changing antibacterial effect of the component drugs, and the bacterial strains involved. Simulations based on these models provide a rational way of predicting differences in effect of clinical dosing regimens of interest and thus suggest regimens for further clinical testing^16,17^.

In this study, we quantified the combination effects of CIP and CST against four *E. coli* strains with varying drug susceptibility through in vitro static time-kill experiments using a PKPD modelling approach. The developed final model comprised of a CST binding model describing drug loss over time due to the adsorption to the lab material and PKPD models for bacterial growth and antibiotic effects describing CIP and CST single drug effect and their interaction. This final model was applied to predict antibacterial effects in human plasma and renal interstitium, representing bloodstream infections and pyelonephritis, respectively, and thereby evaluate the potential clinical benefit of adding a relatively low CST dose to CIP monodrug therapy.

## Results

**Figure 1:**
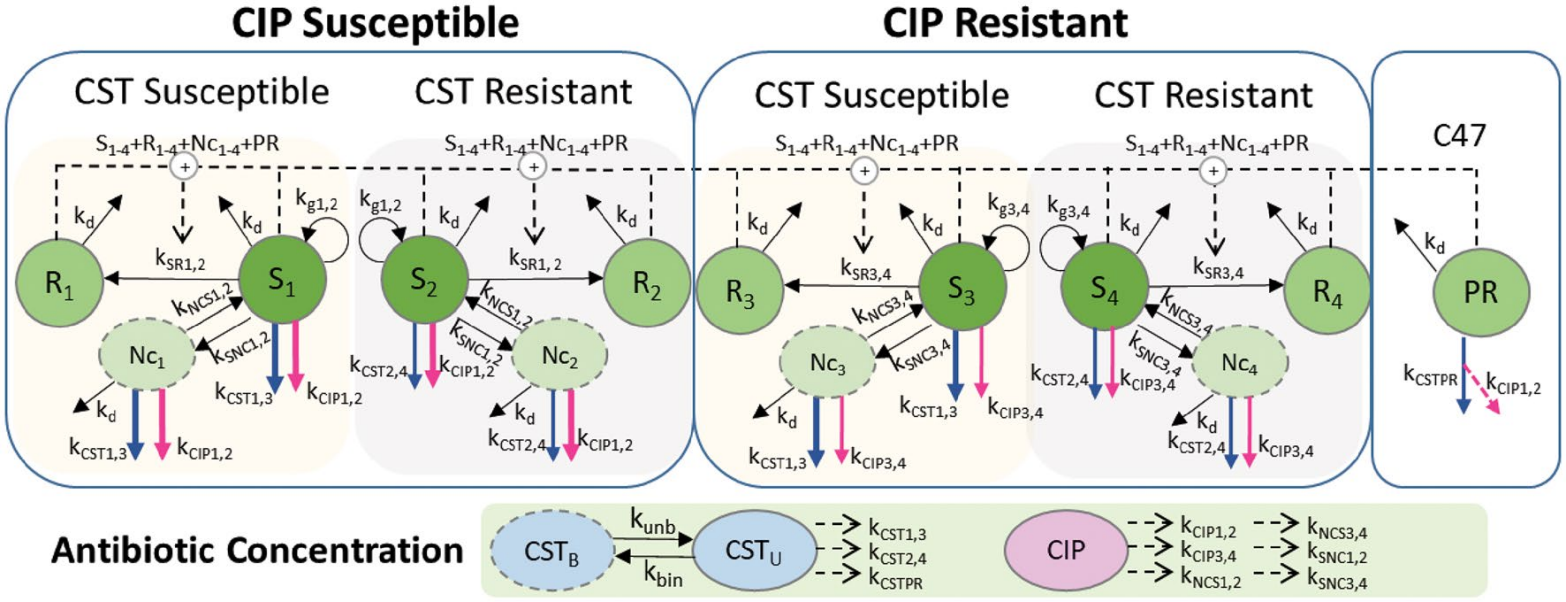
Schematic illustration of the final integrated PKPD model consisting of both the bacterial model (top row) and the antibiotic concentration model (bottom row). The bacteria inoculum was split into five subpopulations: (1) ciprofloxacin (CIP) susceptible and colistin (CST) susceptible; (2) CIP susceptible and CST resistant; (3) CIP resistant and CST susceptible; (4) CIP resistant and CST resistant; and (5) Pre-existing Resting (PR)—only for strain C47. For subpopulations 1–4, bacteria could transfer from a growing S-state to a resting R-state as a response to high population bacterial densities in the system (S_1-4_ + R_1-4_ + Nc_1-4_ + PR) and to and from a transiently non-colony forming Nc-state as response to CIP exposure. Bacteria in S- and Nc- states could be killed by CIP (pink arrows) and CST (blue arrows) with different rates dependent on the susceptibility. PRs are non-growing bacteria that could not be killed by CIP but by CST. They resumed CIP susceptibility when CST was co-administered. The killing effect was exerted by the unbound CST (CST_U_), predicted by a binding model, while the concentrations of effective CIP remained nominal. Their respective concentration-dependent rate constants are listed beside. See Table 1 for description of all parameter estimates.

The final integrated PKPD model (Fig. 1) included a component characterizing the CST concentrations in the experimental system, constant CIP concentrations, as well as components for bacterial growth and antibiotic effects that linked the drug concentration–time profiles to the effect on the bacteria.

### CST concentration models

CST has been reported to bind to glass and plastic lab material^18^. Therefore, it is important to determine the actual concentrations in the experimental system. The CST binding to test tubes was considered by describing the free fraction (*fu*) of CST at the start of the experiments by an Emax function. *fu* was predicted to range from 0.40 to 0.96 for nominal concentrations of 0.0625 to 8 mg/L. The labware binding kinetics during the experiment was well described by the two-compartment model with Emax binding kinetics and first-order unbinding kinetics. Parameter estimates of these two submodels are listed in Supplementary Table S1. Goodness-of-fit plots (Supplementary Fig. S1 and S2) indicate a reasonable fit of the models.

### PKPD models for bacterial growth and antibiotic effect

**Figure 2:**
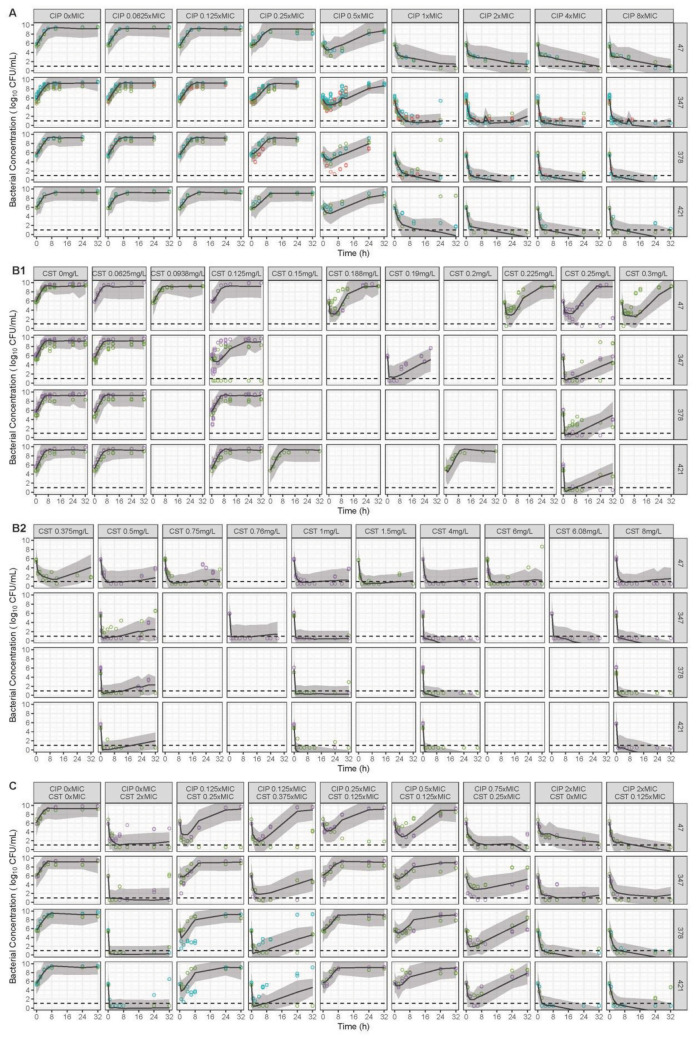
Visual predictive checks based on the final PKPD model for (A) ciprofloxacin (CIP) monodrug; (B1 and B2, consecutively) colistin (CST) monodrug; and (C) CIP and CST in combination. Each plot is stratified by strains (rows, *E. coli* C47, LM347, LM378 and LM421) and nominal drug concentrations (columns, either in xMIC or mg/L). The lower dashed line represents the lower limit of detection (LLOD, 1 log_10_ cfu/mL). The observations are shown in circles, with colors representing different replicates. Observations below LLOD are presented at 0.5 log_10_ cfu/mL. The predictions are shown as median (solid lines) and its 95% confidence interval (grey area) simulated (n = 500) based on the final PKPD model.

Overall, 303 time-kill curves were used to establish the PKPD model. A summary of the number of curves, CFU counts, and experimental days for the whole dataset is presented in Supplementary Table S2. The parameter estimates are listed in Table 1. The fit of the final PKPD model is depicted in a visual predictive check (VPC) stratified on drug, concentrations, and strains (Fig. 2), and shows that the model could satisfactorily capture the trend of bacterial counts over time.

**Table 1:** Parameter estimates and relative standard errors (RSEs) of the final pharmacokinetic-pharmacodynamic (PKPD) model.

**Parameter**	**Explanation**	**Value (RSE**^**1**^**)**		
***Bacterial growth model***				
*k*_*g1,2*_ (/h)	Growth rate constant for subpopulations 1, 2	1.69 FIX^2^		
*k*_*g3,4*_ (/h)	Growth rate constant for subpopulations 3, 4	0.316 FIX^2^		
*k*_*d*_ (/h)	Natural death rate constant	0.179 FIX^2^		
*Bmax* (cfu/mL)	Maximum bacterial density in the system	1.56 × 10^9^ (11%)		
***CIP monodrug model***^2^				
${E}_{maxCIP}$(/h)	${E}_{max}$ for ${k}_{CIP\mathrm{1,2}}$ and ${k}_{CIP\mathrm{3,4}}$	6.75 FIX^2^		
${EC}_{50CIP,\mathrm{1,2}}$(mg/L)	${EC}_{50}$ for ${k}_{CIP\mathrm{1,2}}$	1.38 × MIC^0.996^ FIX^2^		
${EC}_{50CIP,\mathrm{3,4}}$(mg/L)	${EC}_{50}$ for ${k}_{CIP\mathrm{3,4}}$	1.53 FIX^2^		
${\upgamma }_{CIP}$	Hill factor for ${k}_{CIP\mathrm{1,2}}$ and ${k}_{CIP\mathrm{3,4}}$	1.60 FIX^2^		
${f}_{MUTCIP}$(/10^6^)	Fraction of CIP resistant bacteria in inoculum	1.23 FIX^2^		
${f}_{MUTP}$(/10^6^)	Fraction of PR bacteria in inoculum for strain C47	3910 FIX^2^		
${k}_{SNc,max}$(/h)	${E}_{max}$ for ${k}_{SNc}$	4.69 FIX^2^		
${t}_{r50}$	${EC}_{50}$ for ${k}_{SNc}$	0.183 FIX^2^		
${\upgamma }_{SNc}$	Hill factor for ${k}_{SNc}$	20 FIX^2^		
${k}_{NcS}$(/h)	Rate constant of transfer from Nc to S	0.449 FIX^2^		
${t}_{Nc}$(h)	Time point when transfer from S to Nc stops	3.2 FIX^2^		
***CST monodrug model***		**C47**	**LM347**	**LM378, LM421**
${E}_{maxCST,\mathrm{1,3}}$(/h)^3^	${E}_{max}$ for ${k}_{CST1,3}$	50 FIX^3^		
${E}_{maxCST,\mathrm{2,4}}$(/h)	${E}_{max}$ for ${k}_{CST2,4}$	1.44 (1.1%)	2.44 (1.1%)^4^	
${E}_{maxCST,PR}$(/h)	${E}_{max}$ for ${k}_{CST,PR}$	2.44 (1.1%)^4^	NA	
${EC}_{50CST}$(mg/L)	${EC}_{50}$ for ${k}_{CST\mathrm{1,3}}$ and ${k}_{CST\mathrm{2,4}}$	0.110 (1.4%)		
${EC}_{50CST,PR}$(mg/L)	${EC}_{50}$ for ${k}_{CST,PR}$	0.251 (16%)	NA	
${\upgamma }_{CST,\mathrm{1,3}}$	Hill factor for ${k}_{CST\mathrm{1,3}}$	5.26 (1.6%)^4^	3.55 (2.0%)	20 FIX^3^
${\upgamma }_{CST,\mathrm{2,4}}$	Hill factor for ${k}_{CST\mathrm{2,4}}$	5.26 (1.6%)^4^	0.270 (5.7%)	
${\upgamma }_{CST,PR}$	Hill factor for ${k}_{CST,PR}$	5.26 (1.6%)^4^	NA	
${f}_{MUTCST}$(/10^6^)	Fraction of CST resistant bacteria in inoculum	3.80 (1.7%)		
***CIP and CST combination model***		**C47**	**LM347**	**LM378, LM421**
I_CST_	Factor on EC_50_ for ${k}_{CST\mathrm{1,3}}$ and ${k}_{CST,PR}$	0.541 (1.3%)^4^	1.19 (3.2%)	0.541(1.3%)^4^
I_CIP_	Factor on EC_50_ for ${k}_{CIP\mathrm{1,2}}$	1.60 (0.75%)		
***Residual model***				
Residual error (log_10_ CFU/mL)		1.29 (3.2%)		
Replicate residual error (log_10_ CFU/mL)		0.0144 (2.7%)		

CIP, ciprofloxacin (${C}_{CIP}$, CIP concentration (mg/L)); CST, colistin; MIC, minimum inhibitory concentration; NA, not applicable; ${E}_{max}$, maximum drug killing rate constant; ${EC}_{50}$, concentrations resulting in half ${E}_{max}$. Parameters listed in the table were shared among strains unless specified with vertical column separator.

^1^RSE, relative standard error, calculated by PsN sampling importance resampling (SIR) tool.

^2^The parameters were fixed to the reported value from CIP monodrug PKPD model^19^.

^3^The parameters were fixed to stabilize the model.

^4^The parameters shared their values to simplify the model but were displayed separately limited by the table layout.

#### General model structure on bacterial growth

The PKPD model described four pre-existing subpopulations with different antibiotic susceptibility: susceptible to both CIP and CST (subpopulation 1), susceptible to CIP and resistant to CST (subpopulation 2), resistant to CIP and susceptible to CST (subpopulation 3), and resistant to both antibiotics (subpopulation 4). For the clinical strain C47, an additional pre-existing resting state (PR) subpopulation, as was originally defined in the CIP monodrug model^19^, was needed to describe the data. Bacteria in the start inoculum (at 0 h) were divided into one of five subpopulations with fractions ${f}_{1-4}$ and ${f}_{PR}$ described by Eqs. (1–5),

1 $${f}_{1}=\left(1-{f}_{MUTCIP}\right)\times \left(1-{f}_{MUTCST}\right)\times \left(1-{f}_{MUTPR}\right)$$

2 $${f}_{2}=\left(1-{f}_{MUTCIP}\right)\times {f}_{MUTCST}\times \left(1-{f}_{MUTPR}\right)$$

3 $${f}_{3}={f}_{MUTCIP}\times \left(1-{f}_{MUTCST}\right)\times \left(1-{f}_{MUTPR}\right)$$

4 $${f}_{4}={f}_{MUTCIP}\times {f}_{MUTCST}\times \left(1-{f}_{MUTPR}\right)$$

5 $${f}_{PR}={f}_{MUTPR}$$

Together, the fractions sum up to 1. ${f}_{MUTCIP}$, ${f}_{MUTCST}$, and ${f}_{MUTPR}$ are the fractions of the bacteria belonging to CIP resistant, CST resistant, and PR subpopulations, respectively. ${f}_{MUTCIP}$ and ${f}_{MUTPR}$ were set to the values reported before^19^. While ${f}_{MUTCIP}$ was set to 1.23 per 10^6^ bacteria and was shared among all strains, ${f}_{MUTP}$ was set to 3910 per 10^6^ bacteria for strain C47 and 0 for the other three strains (LM347, LM378, and LM421) indicating no existing PR subpopulation for the lab strains. ${f}_{MUTCST}$ was estimated to 3.8 per 10^6^ bacteria in the CST monodrug model. To note, the bacterial density in subpopulation 4, i.e. for bacteria resistant to both CIP and CST, never exceeded 1 CFU/mL in investigated scenarios but was included for completeness, as theoretically a subpopulation that is resistant to both drugs could exist.

For subpopulations 1 to 4, bacteria started in the susceptible (S) state and moved to the resting and non-growing (R) state or transiently to and back from the non-colony forming yet drug-susceptible (Nc) state according to the differential Eqs. (6–8),

6 $$dS/dt=\left({k}_{g}-{k}_{d}-{k}_{Drug }-{k}_{SR}-{flag}_{Nc}\times {k}_{SNc}\right)\times S+{k}_{NcS}\times Nc$$

7 $$dR/dt= -{k}_{d}\times R+{k}_{SR}\times S$$

8 $$dNc/dt= \left(-{k}_{d}-{k}_{Drug }-{k}_{NcS}\right)\times Nc+{flag}_{Nc}\times {k}_{SNc}\times S$$

where the initial condition for S is the fractional inoculum ($=\text{Inoculum Size}\times {f}_{1-4}$, see Eqs. 1–4) and 0 for R and Nc. These equations have earlier been presented when the CIP monodrug PKPD model was developed^20^. In short, ${k}_{g}$ and ${k}_{d}$ represent bacterial natural growth and death rate constants, respectively, in the absence of antibiotics. ${k}_{g}$ was determined to be lower for the CIP resistant subpopulation, i.e. a fitness cost, and the bacterial doubling time $\left(={\text{ln}}2/\left({k}_{g}-{k}_{d}\right)\right)$ were 28 min and 5.1 h for CIP susceptible and resistant subpopulations, respectively^19^. ${k}_{Drug}$ represents the antibacterial killing rate constant and is detailed below. ${k}_{SR}$ is the rate constant of bacterial transfer from S to R state and was dependent on the estimated maximal bacterial density (${B}_{max}$) and real-time overall bacterial density in the system (${B}_{TOT}$) according to Eqs. (9) and (10),

9 $${k}_{SR}=\left({k}_{g}-{k}_{d}\right)\times {B}_{TOT}/{B}_{max}$$

10 $${B}_{TOT}=S1+{\text{R}}1+{\text{Nc}}1+{\text{S}}2+{\text{R}}2+{\text{Nc}}2+{\text{S}}3+{\text{R}}3+{\text{Nc}}3+{\text{S}}4+{\text{R}}4+{\text{Nc}}4+{\text{PR}}$$

In addition, bacteria could transfer between S and Nc states (${k}_{SNc}$ and ${k}_{NcS}$) in a CIP concentration-dependent manner^20^. The transfer rate ${k}_{SNc}$ was estimated to shut off after 3.2 h^19^, as governed by the indicator ${flag}_{Nc}$. PRs changed over time according to Eq. (11), with initial condition $=\text{Inoculum Size}\times {f}_{PR}$ (see Eq. 5), and these bacteria could not multiply or move to other states.

11 $$dPR/dt=\left(-{k}_{d}-{k}_{Drug }\right)\times PR$$

#### Single drug effect model

As illustrated in Fig. 1, ${k}_{Drug}$ was equal to ${k}_{CIP\mathrm{1,2}}$ and ${k}_{CIP\mathrm{3,4}}$ for CIP susceptible and resistant subpopulations, respectively, under CIP monodrug exposure. Similarly, ${k}_{Drug}$ was ${k}_{CST\mathrm{1,3}}$ and ${k}_{CST\mathrm{2,4}}$ for CST susceptible and resistant subpopulations, respectively, under CST monodrug exposure. These rate constants were described by sigmoid Emax functions (Eq. 12) with parameter estimates listed in Table 1.

12 $${k}_{Drug}={E}_{max}{\times C}_{Drug}^{\gamma }/\left({EC}_{50}^{\upgamma }{+C}_{Drug}^{\gamma }\right)$$

where ${E}_{max}$ is the maximum killing rate constant, ${EC}_{50}$ is the antibiotic concentration (${C}_{Drug}$) at half ${E}_{max}$, and $\gamma$ is the sigmoid factor. While ${k}_{CIP}$ shared ${E}_{max}$ and $\gamma$ among strains and subpopulations and included a power function between ${EC}_{50}$ and the minimum inhibitory concentrations (MICs) for the susceptible subpopulations (${EC}_{50CIP, \mathrm{1,2}}$), ${k}_{CST}$ shared ${EC}_{50}$ and was estimated to 0.11 mg/L. The *E*_*max*_ of CST susceptible subpopulations (${E}_{maxCST, \mathrm{1,3}})$ was shared among all strains and fixed to 50/h (i.e. minimum half-life of ~ 1 min). This value was selected in a sensitivity analysis (by evaluating the model fit when the parameter was fixed to 30, 50, 100, and 150) and 50 was the lowest possible value without compromising the model fit while stabilizing the model. Estimated ${E}_{max}$ values for the colistin-resistant subpopulation, i.e. ${E}_{maxCST, \mathrm{2,4}}$, were only 3–5% of ${k}_{CST1}$ (i.e. minimum half-life of ~ 20–30 min). The estimated ${\gamma }_{CST}$ values indicated the steepness of the concentration-effect curves and differed among strains and subpopulations. The PR subpopulation was resistant to CIP under monodrug exposure^19^. The estimated CST effect (${k}_{CSTPR}$) was described by a sigmoid Emax function (Eq. 12, see Table 1).

#### Drug combination effect model

A simple additive killing effect without interaction terms, i.e. ${k}_{Drug }= {k}_{CIP}+{k}_{CST}$ for respective subpopulation, adequately fit most of the observed data under the combined exposure of CIP and CST, indicating subpopulation synergy^17^. However, inclusion of the interaction terms I_CIP_ and I_CST_ (Table 1) on the drug potency parameter EC_50_ improved the model fit significantly (*d*OFV = 551, *df* = 3, p < 0.001). Both I_CIP_ and I_CST_ were concentration-independent factors in the studied concentration range. The estimated I_CIP_ of 1.6 indicated that the CIP potency was nearly halved for all strains when CST was present as ${EC}_{50CIP,\mathrm{1,2}}$ was 160% of the value under CIP monodrug exposure. The CST potency was however estimated to be doubled when combined with CIP (${EC}_{50CST}$ was 54.1% of the value under CST monodrug), except for the wild-type strain LM347, where the CST potency was decreased (${EC}_{50CST}$ increased by 119%). When the interaction terms were added on E_max_ instead of EC_50_, the model fit was almost as good (*d*OFV = 7, *df* = 0, AIC criteria, i.e. the lower OFV the better). I_CIP_ and I_CST_ were only supported for susceptible subpopulations (${k}_{CIP\mathrm{1,2}}$ and ${k}_{CST\mathrm{1,3}}$) but not for resistant subpopulations (${k}_{CIP\mathrm{3,4}}$ or ${k}_{CST\mathrm{2,4}}$). For the bacteria in subpopulation P, the best model indicated that CIP regained its antibacterial ability when co-administrated with CST (*d*OFV = 20, *df* = 0, AIC criteria, i.e. the lower OFV the better): ${k}_{Drug }={k}_{CIP\mathrm{1,2}}+{k}_{CSTPR}$ with I_CIP_ and I_CST_ being included on their respective EC_50_ as described above.

### Prediction of clinical drug effect

**Figure 3:**
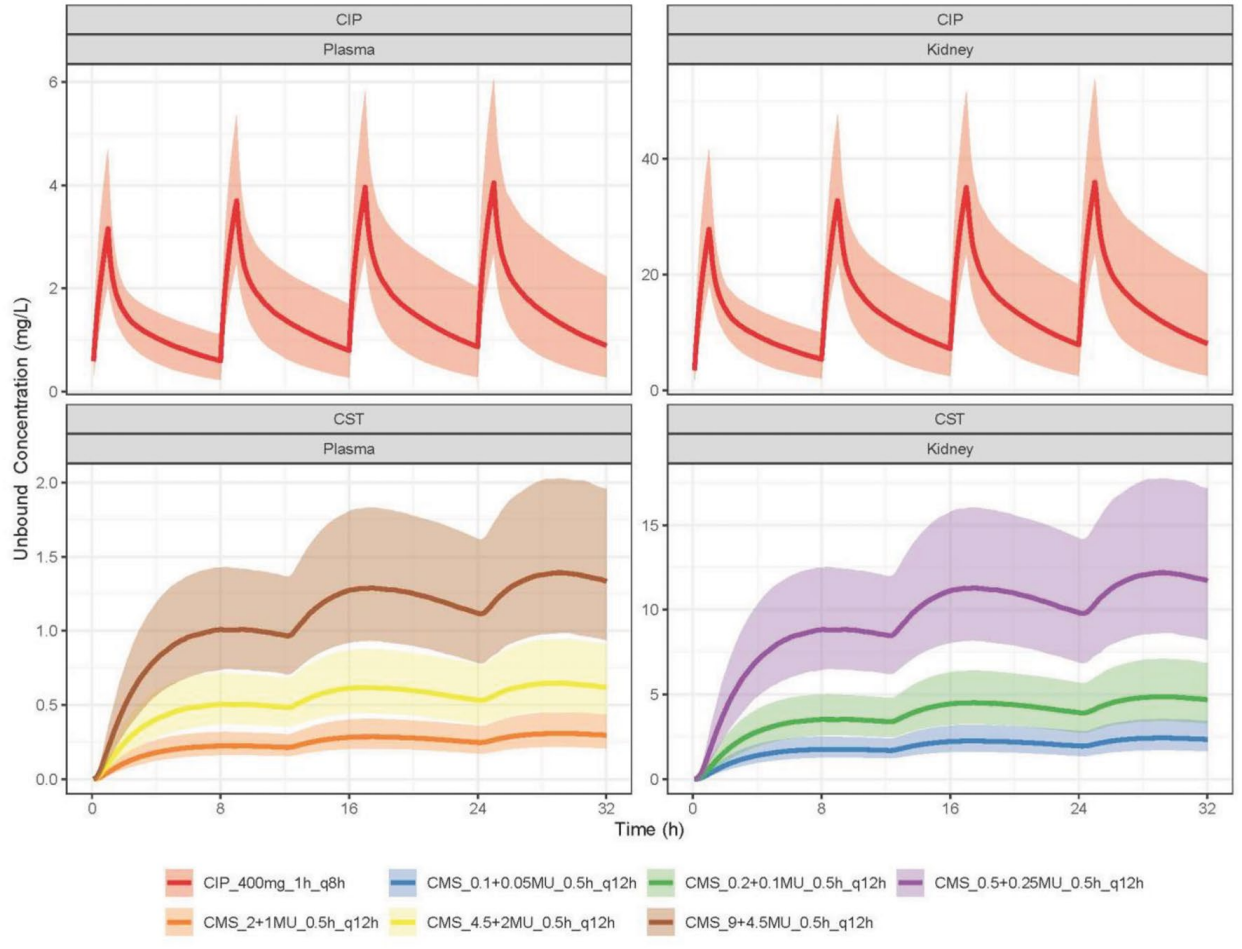
Simulated unbound concentrations over time for ciprofloxacin (CIP) or colistin (CST) in plasma or interstitium in kidney under various doses. The doses are expressed in the format Drug_Dose (Loading + Maintance)_InfusionTime_Frequency. For example, CIP_400mg_1h_q8h indicates that CIP was administered at a dose of 400 mg every 8 h, with each dose infused over a period of one hour. CMS_9 + 4.5MU_0.5_q12h indicates that CMS (the prodrug of CST) was administered at a dose of 9MU loading dose plus 4.5MU maintenance dose every 12 h, with each dose infused over a period of 0.5 h. The predictions are shown as median (solid lines), and its 80% prediction interval (shaded area) of 1000 simulated patients.

The unbound antibiotic concentrations predicted in plasma and kidney interstitium under the simulated dosages are shown in Fig. 3. The kidney to plasma concentration ratio was predicted to be around 10 and 150 for CIP and CST, respectively. Consequently, a more effective bacterial killing is expected for pyelonephritis than for blood stream infections. Therefore, higher MICs and lower antibiotic doses than used for bloodstream infections were adopted in the simulations to investigate a potential benefit of the drug combination.

**Figure 4:**
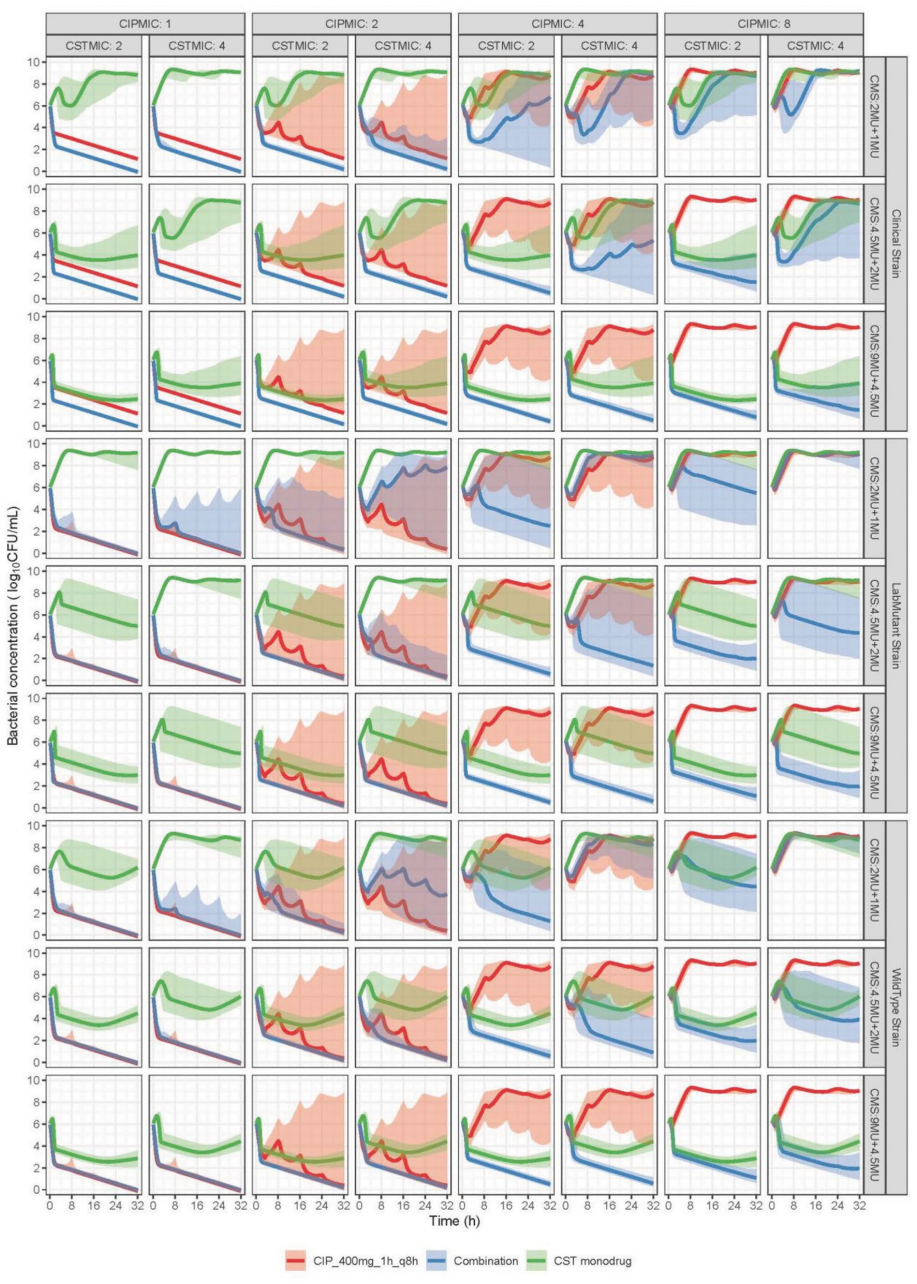
Simulated bacterial loads over time in plasma, faceted by strains from different sources (clinical strain, wildtype strain, or lab mutant strain) shown in rows, and by different MIC values (CIPMIC and CSTMIC in mg/L) shown in columns. Antibiotics were administered at a bacterial concentration of 6 log10 CFU/mL. Ciprofloxacin (CIP) dose was 400 mg infused every 8 h intravenously for 1 h in all cases. Colistimethate sodium (CMS) doses, as specified in the facet labels for each row, were either a 9 MU loading dose followed by a 4.5 MU maintenance dose (abbreviated as CMS:9MU + 4.5MU), or 4.5MU + 2MU, or 2MU + 1 MU, infused every 12 h intravenously for 30 min, either as a single drug or in combination. The predictions are shown as median (solid lines), and its 80% prediction interval (shaded area) of 1000 simulated patients.

**Figure 5:**
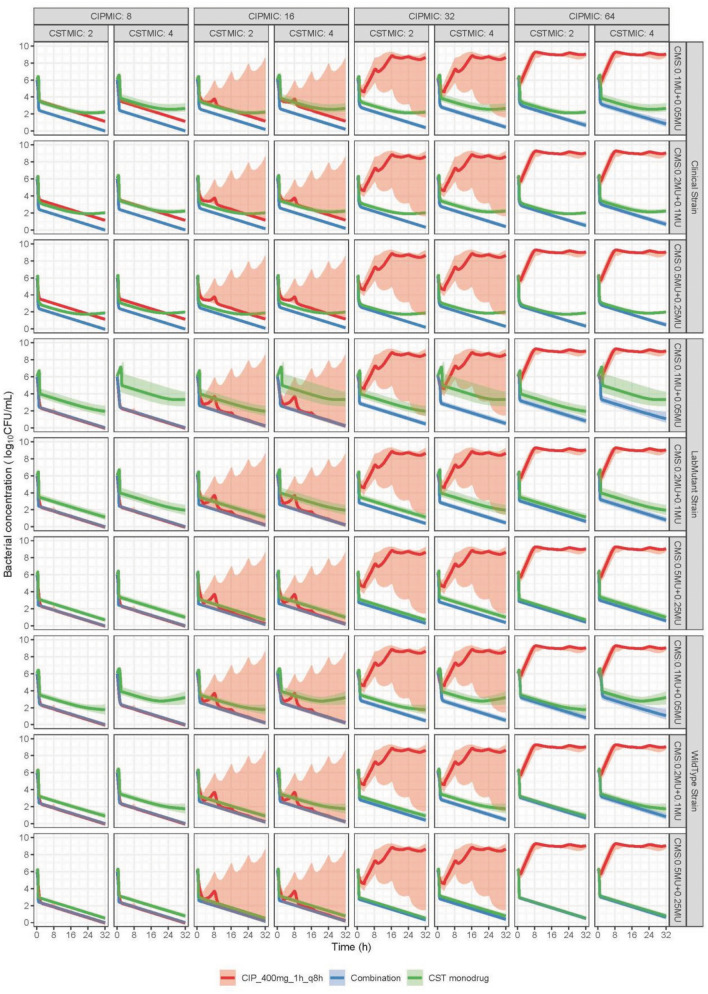
Simulated bacterial loads over time in kidney, faceted by strains from different sources (clinical strain, wildtype strain, or lab mutant strain) shown in rows, and by different MIC values (CIPMIC and CSTMIC in mg/L) shown in columns. Antibiotics were administered at a bacterial concentration of 6 log10 CFU/mL. Ciprofloxacin (CIP) dose was 400 mg infused every 8 h intravenously for 1 h in all cases. Colistimethate sodium (CMS) doses, as specified in the facet labels for each row, were either a 0.5 MU loading dose followed by a 0.25 MU maintenance dose (abbreviated as CMS:0.5MU + 0.25MU), or 0.2MU + 0.1MU, or 0.1MU + 0.05MU, infused every 12 h intravenously for 30 min, either as a single drug or in combination. The predictions are shown as median (solid lines), and its 80% prediction interval (shaded area) of 1000 simulated patients.

The predicted bacterial counts over time in plasma and kidney interstitium are shown in Fig. 4 and 5,respectively. In general, the superiority of the combination is dependent on the administered antibiotic doses, strain susceptibility and the infected site. The delay in formation of CST from CMS (Fig. 3) resulted in an increase in bacterial load in the beginning of CST monotherapy. The predicted monophasic decline after 8 h for CST monotherapy was in line with the absence of regrowth at high CST concentrations in the in vitro experiments (Fig. 2B2). In plasma, a given colistin methanesulfonate (CMS, prodrug of colistin) 9 MU loading dose (LD) plus 4.5 MU maintenance dose (MD) q12h was predicted to result in larger or equivalent reduction in bacterial concentrations compared to either CST or CIP alone in all scenarios, even though some overlap of the prediction interval was observed for monodrug and the combination at the highest target MICs (MIC_CIP_ = 8 mg/L, MIC_CST_ = 4 mg/L). When the CMS dose was halved to 4.5 MU LD plus 2 MU MD q12h (simplified as 4.5 + 2 hereafter), the superiority of the combination could be concluded for the strains with MIC_CST_ of 2 mg/L but not for the ones with MIC_CST_ of 4 mg/L. For the latter, the prediction intervals of bacterial concentrations under combination widely overlapped with that under monotherapy, indicating the combination may be effective for a typical (median) individual but not sufficiently efficacious in all patients. A further reduction of the CMS dose to 2 + 1 showed no improvement of the combination over monodrug for any of the simulated strains. For the clinical strain, the combination therapy was predicted to result in a higher (dose-independent) killing effect compared to CIP as monodrug, since being also effective against the PR subpopulation.

In kidney interstitium, since CMS doses above 0.5 + 0.25 resulted in maximum bacterial killing rate under monotherapy, the illustrated doses in the figures are at 0.5 + 0.25 or lower. The lower the CMS dose, the larger was the additional antibacterial effect compared to monodrug. The typical predicted bacterial counts diminished to < 10 CFU/mL. Similar to the results in plasma, the combined effect was always superior to CIP alone for clinical strains as CST could kill PRs.

## Discussion

In this study, we developed a semi-mechanistic PKPD model describing the antibacterial effect of CIP and CST as monodrug and in combination against four *E. coli* strains with different susceptibilities to CIP. The final model (Fig. 1), apart from components for antibiotic concentrations changing over time, consisted of up to five pre-existing subpopulations with different susceptibility to the two antibiotics. Bacteria resided in the four CIP and CST susceptible or resistant (2 × 2) subpopulations transferred between S, R and Nc states in response to the drug concentration and bacterial load in the system. The clinical strain C47 had one additional PR subpopulation which was fully resistant to CIP, susceptible to CST and regained susceptibility to CIP when CST was co-administrated. The PKPD model quantified the combination effect as an alteration in drug effect potency (EC_50_) in addition to the subpopulation synergy. The direction of the alteration was strain- and drug-dependent, and model predictions/simulations are needed to understand the impact of the combination on bacterial killing over time and to provide a visualization of the clinical potential of the combination therapy.

The simulations conducted here illustrated an example of applying a PKPD model in predicting and comparing potential clinical dosing regimens^21^. Moreover, our study expanded the conventional focus on bloodstream infections by also illustrating the simulation at the target site of infection using interstitial concentrations predicted from WB-PBPK models^22,23^. Overall, our simulation illustrated the potential efficacy of the combining CIP with a lower-than-commonly-administrated CST dose in killing bacteria compared to either drug alone, yet the extent depended on the administrated dosages, the source of the strains, their susceptibility to the drugs, and the site of infections. In plasma, a higher than monodrug killing effect when co-administrating CIP at half of the normal CST dosage, i.e. CMS 4.5 MU LD plus 2 MU MD q12h, was predicted for simulated strains with MIC_CIP_ = 1–8 mg/L and MIC_CST_ = 2 mg/L. In kidney interstitium, the drug concentrations were predicted to be higher, and consequently drug effects seen at even lower doses.

Notably, the strains used in the simulation were more resistant than those used to develop the PKPD model, aiming to better approximate the clinical scenarios where combination therapy would be a potential choice for clinicians. It should be noted that the extrapolation to untested theoretical bacteria was conducted by implementing postulated EC_50_-MIC relationships for CST derived from the CIP monodrug model.WB-PBPK models allow for prediction theoretically at any potential physical site of interest. The models we adopted^22,23^ enabled prediction of unbound concentrations in kidney interstitium. However, the predictions were dependent on the assumptions made in the WB-PBPK model and the parameters used (detailed in Supplementary methods), and could not be verified due to a lack of experimentally measured concentrations. It should be acknowledged that urine concentration is not likely to be the same as unbound kidney concentration, especially for CST that accumulates in kidney (causing nephrotoxicity) and is only excreted in the urine to a limited extent^24^. The large predicted (10- or 150-fold) differences between unbound kidney and plasma concentration in our study is consistent to that reported from WB-PBPK models^22,23^. It also agrees with the experimentally observed accumulation and slow elimination of CST in pigs^25^, and of CIP in humans^26^. Nevertheless, further studies are needed to understand how the concentrations in kidney translate to the bacterial killing effect. Our simulations are primarily intended to illustrate how predicted unbound concentrations at the site of infection can be of value to predict bacterial kill of various dosages for different indications, and how drug combinations can be explored before clinical studies are initiated.

A pre-existing subpopulation (i.e. heteroresistance) model was here determined to be more favorable than an adaptive resistance model structure for the drug effect of CST drug effect. This was in part supported by the observed (and genetically confirmed) selection of heteroresistant subpopulations in the here used wild type *E. coli* (LM347) at both high and low CST concentrations^27^. Mechanistically, both heteroresistance and adaptive resistance may however occur for CST. The concurrent presence of these two phenotypic traits in the same strain has earlier been suggested^28,29^, and both model structures have been successfully applied to describe polymyxin effects without a clear pathogen-dependent preference^30–34^. In terms of parameters, the finding that EC_50_ but not E_max_ could be shared between susceptible and resistant subpopulations for CST is consistent with an earlier observation that heteroresistant bacteria differed from the corresponding dominant populations by E_max_ only, i.e. there was no difference in their EC_50_^34^. That EC_50_ could be a shared parameter for the four different strains is in line with their similar MIC_CST_ values (0.5–0.75 mg/L).

The interaction terms in the drug combination model were concentration-independent and showed similar fit when included for EC_50_ as for Emax, which could suggest that the identification of the underlying mechanisms is not supported by the available data. This might be because the subpopulation synergy model is inherently concentration-dependent, and the data could not support further complexity. Further studies are needed to mechanistically understand the estimated interaction coefficients, which were here data-driven. Additionally, the combination model indicated that co-administered CST could “revive” the susceptibility to CIP of the PRs, which are the potentially remnants from stationary phase cultures^35^. However, the mechanistic validity of this data-driven hypothesis requires further investigation.

A limitation of this study was the variation in observed CFU counts in time-kill experiments. This likely reflects both biological and experimental variability given that the experiments were conducted at different sites (labs), by several technicians, and spanned over a few years, despite that the same protocol was used. In our final model, we focused on the general trends of CFU over time, which was reasonably well captured despite the variability. We used one creatinine clearance (CrCL) value (90 mL/min) in the simulations and acknowledge that simulated variability may be lower than the expected in a patient population with a wide range of CrCL values. Our simulations were however made to showcase the approach rather than to make dose recommendations based on kidney function.

To conclude, in this study, we quantified CIP and CST effects as monodrugs and as a combination against four strains of *E. coli* by establishing an in silico PKPD model. This model enabled us to simulate the time-course of bacteria in human plasma and interstitium of kidney upon antibiotic exposure and to explore the benefit of the combination by lowering the doses of the more toxic component (CST), for different strains. Our study provides a valuable framework of modelling and simulating antibiotic combination effects, showing the potential application of a PKPD model in predicting the combination effect at clinical infection sites of interest.

## Materials and methods

### Antibiotics, material, strains and MIC‐determination

CIP and CST (colistin sulphate) were purchased from Sigma Aldrich (Schnelldorf, Germany). Stock solutions (10 mg/mL) were prepared in PBS for CST and 0.1 M HCL for CIP. To minimize the loss of colistin due to binding to lab material^18^, a fixed protocol for dilutions was followed to minimize the number of dilution steps to two to four depending on targeted colistin concentration. Solutions were diluted to 1, 10, 100 or 1000 ug/mL using PBS and then further diluted using cation-adjusted Mueller Hinton II (CAMHII) broth (BD BBL™ reference number 212322) to the desired concentrations. Falcon® tubes (reference number 352096) were used in all work with colistin.

Four *E. coli* strains with distinct phenotypic susceptibility towards CIP were used in this study: one MG1655 wildtype lab strain (LM347; MIC_CIP_ 0.023 mg/L and MIC_CST_ 0.5 mg/L), two isogenic mutant lab strains (LM378 with gyrA1 S83L and LM421 with gyrA1 S83L plus marR knockout; MIC_CIP_ 0.38 and 1.0 mg/L, respectively, and MIC_CST_ 0.5 mg/L for both) and one clinical isolate (C47, originating from a patient with uncomplicated urinary tract infection, MIC_CIP_ 0.047 mg/L, MIC_CST_ 0.75 mg/L). The MICs were determined by macrodilution in CAMHII broth^20^.

### Time-kill experiments

CIP monodrug data were from a previously published dataset^19^. Here only the data from the four strains used here and a standard start inoculum (~ 10^6^ CFU/mL) were utilized. CST monodrug and combination data were collected in experiments dedicated to this study and performed in at least duplicate and conducted in different labs. For CST monodrug, the selected concentrations were 0.125 -16 xMIC (plus growth control) and the sampling time points were 0, 1, 2, 4, 6, 9, 12, 24 and 32 h after adding the antibiotics. For the combination, the concentrations and time points were selected based on optimal design (in PopED) using a preliminary PKPD-model developed for two strains: C47 and LM347^36^. The selected concentrations were CIP 0.125–0.75 xMIC and CST 0.125–0.375 xMIC in combination plus monodrug control (at 2 xMIC) and growth control. The selected sampling time points were 0, 0.75, 2, 4, 6, 8, 24 and 32 h.

From single colonies, overnight cultures (15–17 h at 37 °C) were prepared in 2 mL CAMHII broth and then diluted 1:100 into 2 mL volume. After 1.5 h when the culture had reached log phase (Optical Density 600 = 0.1–0.3, approximately 10^8^ CFU/mL), a second dilution (1:100 into 2 mL) was made to achieve a start inoculum of around 10^6^ CFU/mL. Antibiotics were added immediately after the first sample was taken. Samples were plated on room tempered CAMHII agar plates (BD BBL™ reference number 211438) and incubated over night at 37 °C for viable count.

### CST concentration measurements

Free CST concentrations at 0 h, i.e. immediately after the addition of CST to the time-kill tubes, were measured in all tubes except for those from the first four CST monodrug experiments, where concentration measurements were not initially designed. A 100 μL sample was drawn and added to 100 μL serum and stored at -70 °C until assayed. In the work-up procedure the thawed sample was mixed with acetonitrile containing 0.1% trifluoroacetic acid (TFA), vortexed for about 5 s and centrifuged for 3 min at 10,000 rpm. 100 μL of the supernatant was added to 150 μL 0.03% TFA in water. They were stored at 4 °C before being loaded in the LC–MS/MS^37^. The limit of quantification (LOQ) was 19.4 ng/mL for CST A and 10.5 ng/mL for CST B, with coefficient of variation (CV%) < 6.2% and the bias <  ± 12.6%^37^. The measured CST concentration was calculated as the sum of CST A and B.

### PKPD model building

The PKPD modelling procedure was conducted stepwise and is described in the following sections sequentially. In short, we first developed CST binding models to acquire in vitro concentration–time profiles, which were utilized in the subsequent PKPD modelling. Thereafter, a previously published model on CIP as monodrug^19^ was extended to describe the time-kill experiment data for CIP and CST, both as single drugs and in combination. This step could be further divided sequentially into (1) updating bacterial growth model using all available growth control data, (2) developing CST monodrug model using all except for drug combination data, (3) developing combination model using all data, and (4) finally refining the model by re-estimating all parameters (except for the ones for CIP monodrug) simultaneously. In all these substeps, the CIP monodrug structure was kept unchanged and parameters were fixed to the reported values^19^ considering they were estimated based on a larger *E. coli* dataset including the four strains studied here.

#### CST concentration models

A model characterizing CST binding to lab material was developed using all 122 samples collected at 0 h. In this dataset, 25.4% of the concentrations were below the LOQ. These samples had nominal concentrations ≤ 0.125 µg/mL. The concentration-dependent free fraction *fu* in the tube was estimated by a (sigmoid) Emax function (Eq. 13):

13 $$fu = fu_{\min } + \left( {fu_{\max } - fu_{\min } } \right) \times CST_{N}^{{\gamma_{fu} }} /(CST_{N}^{{\gamma_{fu} }} + fu_{c50}^{{\gamma_{fu} }} )$$

where CST_N_ is the nominal CST concentration at 0 h. The estimated parameters were the baseline and maximum *fu* (*fu*_min_ and *fu*_max_), the CST_N_ resulting in half *fu*_max_ (*fu*_c50_) and the sigmoid factor (${\gamma }_{fu}$).

The reduction of unbound CST concentrations (CST_U_) over time due to labware binding during the time-kill experiment was described using published longitudinal adsorption data (Fig. 2 in ^18^) where polypropylene tubes were used, and experimental conditions were well controlled. Data were extracted from the plot using *GetData Graph Digitizer 2.26 (*https://getdata-graph-digitizer.software.informer.com/*).* The model included two compartments representing unbound and bound CST (CST_U_ and CST_B_), respectively (Eqs. 14 and 15):

14 $${{dCST}_{U}/dt=-CST}_{U}\times {k}_{binMAX}\times \left({1-CST}_{U}^{{\upgamma }_{B}}/\left({CST}_{U}^{{\upgamma }_{B}}+{B}_{50}^{{\upgamma }_{B}}\right)\right)+{CST}_{B}\times {k}_{unb}$$

15 $${dCST}_{B}/dt={CST}_{U}\times {k}_{binMAX}\times \left({1-CST}_{U}^{{\upgamma }_{B}}/\left({CST}_{U}^{{\upgamma }_{B}}+{B}_{50}^{{\upgamma }_{B}}\right)\right)-{CST}_{B}\times {k}_{unb}$$

Initially (at 0 h), the unbound CST was calculated as $\left({CST}_{U}={{\text{CST}}}_{N}\times fu\right)$, with $fu$ extracted from Fig. 2^18^. The binding rate constant was estimated by a CST_U_-dependent (sigmoid) Emax function where *k*_*binMAX*_ denotes the maximum binding rate constant, B_50_ the CST_U_ that results in half *k*_*binMAX*_, and ${\upgamma }_{B}$ the sigmoid factor. *k*_*unb*_ is the rate constant of CST transferring back from bound to unbound.

In the following PKPD modelling process, the CST binding parameters were fixed to their estimated values. CST_U_ was the CST concentration driving the antibacterial effect in the PKPD model.

#### Re-estimation of bacterial growth model parameters

It was explored if re-estimation of the growth rate constant (k_g_), rather than being fixed to the reported value^19^, would result in a significant improvement. The maximum total bacterial density in the system (Bmax) was estimated.

#### CST monodrug model

To explain the observed resistance development during the CST experiments, different resistance models were tested. Adaptive resistance was first tried but was discarded due to that a high concentration-independent resistance development rate constant was estimated (> 20 /h for the three laboratory strains). The interpretation that bacteria will almost immediately (half-life < 0.05 h) become fully resistant once exposed to CST was deemed mechanistically implausible. Consequently, pre-existing subpopulation models were explored. Since the existing two-subpopulation model for CIP monodrug^19^ was insufficient to describe the CST data in this study, and to be compatible with the CIP monodrug model, each CIP subpopulation was divided into CST susceptible or resistant subpopulations. The proportion of bacteria in the pre-existing CST resistant subpopulation was estimated. It was assumed that bacteria will not move to the Nc state, a non-colony-forming state introduced in CIP monodrug model representing filament formation under CIP exposure, when being exposed to CST alone, as no evidence of filamentation formation under CST exposure could be found. PRs were regarded as a distinct subpopulation keeping the characteristic of being non-growing and non-susceptible to CIP monodrug as initially defined^19^ but susceptible to CST as no biphasic killing behaviour was observed following high CST exposure. The CST killing rate constant (${k}_{CST}$) was estimated using a sigmoid Emax function:

16 $${k}_{CST}={E}_{maxCST}{\times CST}_{U}^{{\upgamma }_{CST}}/\left({EC}_{50CST}^{{\upgamma }_{CST}}{+CST}_{U}^{{\upgamma }_{CST}}\right)$$

${E}_{maxCST}$, ${EC}_{50CST}$ and ${\gamma }_{CST}$ were allowed to be strain and subpopulation specific when there was a significant improvement of the model fit when differences were explored.

#### CST and CIP combination model

A subpopulation synergy without interaction term was first tested when CIP and CST were combined. The bacteria in the Nc state were assumed to be susceptible to CST, with the same rate constant as in the S state. The interaction terms were thereafter tested to enhance or decrease E_max_ and/or EC_50_ values of either CIP or CST monodrug as exemplified in Eq. (17) for a case where interaction terms were added on ${E}_{max}$ of CST and ${EC}_{50}$ of CIP,

$${k}_{Drug}={E}_{maxCST}\times \left(\frac{ICST\times {{C}_{CIP}}^{\gamma ICST}}{{{ICST}_{50}}^{\gamma ICST}+{{C}_{CIP}}^{\gamma ICST}}\right){\times CST}_{U}^{{\gamma }_{CST}}/\left({EC}_{50CST}^{{\gamma }_{CST}}{+CST}_{U}^{{\gamma }_{CST}}\right)+$$

17 $${E}_{maxCIP}{\times {C}_{CIP}}^{{\upgamma }_{CIP}}/\left(({{EC}_{50CIP}\times (\frac{ICIP\times {CST}_{U}^{\gamma ICIP}}{{{ICIP}_{50}}^{\gamma ICIP}+{CST}_{U}^{\gamma ICIP}}))}^{{\upgamma }_{CIP}}+{{C}_{CIP}}^{{\upgamma }_{CIP}}\right)$$

where both terms were sigmoid Emax functions. The Emax functions were simplified when applicable, e.g., to concentration-independent constants. ICST and ICIP were allowed to be of any positive value. For the PRs, it was also tested whether CIP would fully regain its killing effect when CST was co-administered.

### PKPD modelling tools and evaluation methods

In silico PKPD modelling was conducted using NONMEM 7.5.0^38^ with the Laplacian method. Model fit was assessed by the objective function value (OFV) (*P* < 0.001, *d*OFV = 10.83, *df* = 1), with scientific plausibility considered. Akaike information criteria (AIC = OFV + 2p, where p is the number of estimated parameters) was used for non-nested models. The transform-both-sides approach was applied to the data using a log_10_ scale. The M3 method^39^ was used to handle data below the limit of detection (LOD, i.e. 1 log_10_ CFU/mL). The residual error was described by an additive error model, and the L2 data item^40^ was applied to handle correlations among replicate samples plated from the same tube and time point. Models were evaluated by checking observations versus predictions plots and simulation based VPCs. PsN 5.3.0 and Pirana 2.9.9 were used to facilitate NONMEM execution^41^. Rstudio 4.1.2 was used to visualize the results.

### Predictions of clinical drug effect

The final developed PKPD model was linked to population PK (popPK) models developed for critically ill patients^42,43^ to simulate (n = 1000) the drug effect in plasma representing bloodstream infections. Reported inter-individual and inter-occasion variability were included. Adopted unbound fraction in plasma (*f*_u,p_) was 0.65 for CIP^22^ and 0.34 for CST^43^. Kidney was selected as an example of a site of clinical interest. It represented the scenario of pyelonephritis due to *E. coli* infection. Considering that most *E. coli* infections are extracellular, free drug concentrations in the interstitial fluid of tissue at the site of infection was assumed to represent the actual target space^44^. Antibiotic concentrations in the interstitium of the kidney were simulated using whole-body physiologically based pharmacokinetic (WB-PBPK) models^22,23^ connected to the popPK models. Details about the parameters and equations used to derive the free kidney concentration profiles are described in the Supplementary Methods.

The simulations aimed to mimic clinical scenarios where the highest applicable CIP dose (400 mg q8h administered intravenously as a 1 h infusion) was predicted to not be effective enough as a monodrug against infections caused by multidrug resistant bacteria. It was explored if combining with CST would be of benefit in such cases and if a low dose would be sufficient, given the risk for CST-induced nephrotoxicity at higher doses. The bacteria strains used for model building was however not applicable for this scenario since they were sufficiently susceptible to be killed by the monotherapy. Simulated strains with elevated resistance were used (details in next paragraph).

Since bacteria with MIC_CIP_ up to 1 mg/L in plasma or 8 mg/L in kidney could be killed by CIP as monodrug without regrowth up to 32 h for a dosage of 400 mg q8h, only strains with higher MIC_CIP_ were tested in the following simulation to check the benefit of co-administrating with CST. Strains with MIC_CIP_ 1–8 mg/L in plasma and strains with MIC_CIP_ 8–64 mg/L in kidney (MIC increasing two-fold) were simulated. These strains were assumed to have MIC_CST_ of 2 or 4 mg/L, with 2 mg/L being the EUCAST epidemiological cut-off values of resistance. Simulated MIC_CIP_ values were scaled to EC_50_ as implemented in the PKPD model. A similar proportional correlation between MIC and EC_50_, as earlier derived over a wide range of values (MICs of 0.023–48 mg/L) for CIP^19^ was assumed. The power on MIC was however fixed to 1 (0.996 estimated for CIP), as shown in Eq. (18) below,

18 $${EC}_{50\_sim}={EC}_{50\_mod}\times {(\frac{{MIC}_{CS{T}_{sim}}}{{MIC}_{CS{T}_{mod}}})}^{1}$$

where the subscript $sim$ and $mod$ indicate parameters used in simulation and from the model, respectively. That EC_50_ and MIC are proportional is a reasonable assumption given that both parameters represent bacterial susceptibilities. This also agrees with the standard PK/PD index methodology, which assumes that for antibiotics where fAUC/MIC is suggested to be the driver of response, the drug exposure needed for efficacy is proportional to the MIC. As for ciprofloxacin, colistin has been suggested to have bacterial response best related to fAUC/MIC^45^.

The adopted CST doses were from CMS 9 MU LD plus 4.5 MU MD as suggested by the latest guidance^45^ and tapered down to 0.1 MU LD plus 0.05 MU MD, all given q12h as a 0.5 h infusion intravenously. Simulations were conducted for the wild type strain (represented by LM347), lab mutant strains (represented by LM378 and LM421, shared), and the clinical strain (represented by C47) separately, to account for strain-dependent parameter estimates in the final PKPD model. The prediction was conducted using the R package mrgsolve^46^.

### Supplementary Information

Supplementary Information.

## Data Availability

Data file and the final PKPD model files are uploaded as supplementary material.
