# Supplementary material for: Quantifying combined effects of colistin and ciprofloxacin against Escherichia coli in an in silico pharmacokinetic-pharmacodynamic model
Source: Sci Rep. 2024 May 22;14:11706. doi: 10.1038/s41598-024-61518-0 (PMC11111785; doi:10.1038/s41598-024-61518-0)
Supplement: Supplementary file 1 — Supplementary Information. [file 41598_2024_61518_MOESM1_ESM.zip › Supplementary/Cip+Col_supplementary_20240402.docx]

**SUPPLEMENTARY MATERIALS**

Quantifying combined effects of colistin and ciprofloxacin against *Escherichia coli* in an *in silico* pharmacokinetic-pharmacodynamic model

Chenyan Zhao ^1^, Anders N. Kristoffersson ^1^, David D. Khan ^1^, Pernilla Lagerbäck ^2^, Ulrika Lustig ^3^, Sha Cao ^3^, Charlotte Annerstedt ^2^, Otto Cars ^2^, Dan I. Andersson ^3^, Diarmaid Hughes ^3^, Elisabet I. Nielsen ^1^ and Lena E. Friberg ^1,^*

^1^ Dept of Pharmacy, Uppsala University, Uppsala, Sweden

^2^ Dept of Medical Sciences, Uppsala University, Uppsala, Sweden

^3^ Dept of Medical Biochemistry and Microbiology, Uppsala University, Uppsala, Sweden

***** Correspondence: [lena.friberg@farmaci.uu.se](mailto:lena.friberg@farmaci.uu.se); Tel.: +46184714685

**Table S1.** Typical parameter estimates and relative standard errors (RSEs) of the colistin binding model

| **Parameter (unit)** | **Explanation** | **Value (RSE**^1^**)** |
| --- | --- | --- |
| ***Submodel for free fraction factor (fu) at the start of the experiments*** | | |
| *fu*_min_ (100%) | Minimum free fraction | 0.328 (10.7%) |
| *fu*_max_ (100%) | Maximum free fraction | 1 FIX^2^ |
| *fu*_c50_ (mg/L) | Colistin concentration at half *fu*_max_ | 0.522 (18.4%) |
| *Err1* (100%) | Proportional error | 0.167 (10.4%) |
| *Err2* (mg/L) | Additive error | 0.0110 (13.7%) |
| ***Submodel for binding during the experiments*** | | |
| *k_binMAX_*  (/h) | Maximum binding rate constant | 0.189 (5.4%) |
| B_50_ (mg/L) | Colistin concentration at half *k_binMAX_* | 0.0347 (13.5%) |
| $k_{unb}$ (/h) | Rate constant of dissociation | 0.122 (9.5%) |
| *Err* (100%) | Proportional error | 0.028 (15.6%) |

^1^RSE was calculated from NONMEM Sandwich matrix.

^2^Estimation of *fu*_max_ did not improve the model fit.

**Table S2.** Number of time-kill curves, CFU counts and experimental days included for ciprofloxacin, colistin and combination dataset for modelling.

|  | Ciprofloxacin | Colistin | Combination |
| --- | --- | --- | --- |
| Time-kill curves | 129 | 102 | 72 |
| CFU counts | 1976 | 1701 | 1028 |
| Experimental days | 16 | 15 | 8 |

(a)


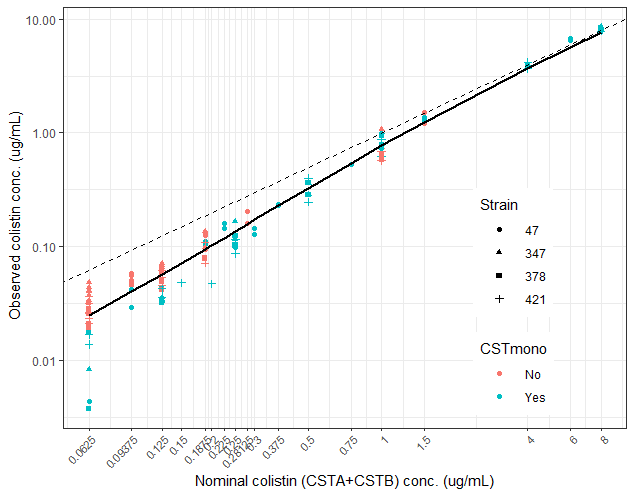


(b)


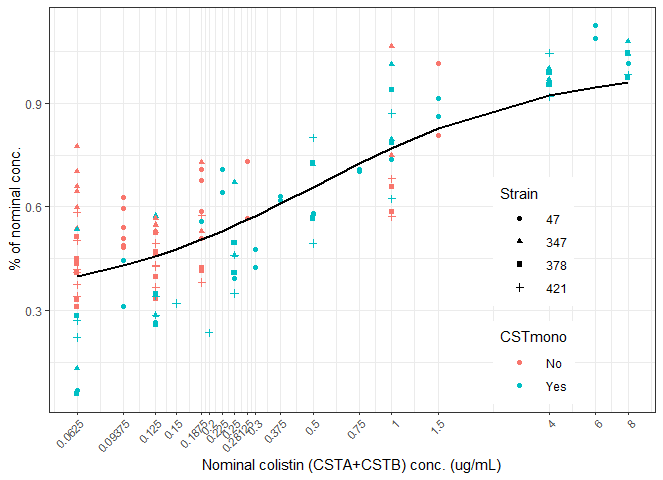


**Figure S1.** Observed colistin concentrations at 0 h expressed (a) in the unit of ug/mL and (b) as percentage of nominal colistin concentration (%), i.e. free fraction (*fu*), versus nominal concentration (ug/mL, sum of measured colistin A and colistin B). The observations are shown as points (shapes indicate strain number and colors indicate whether from mono or combination drug experiments). Model-predicted typical curve is shown as a solid line. Line of identity is shown as a dashed line in (a).


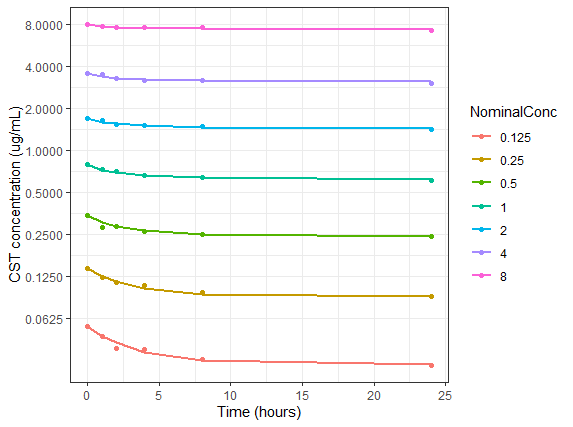


**Figure S2.** Observed (dots) colistin concentrations and model predictions (lines) over time. Colors indicate the nominal concentration levels. Observations were extracted from the published adsorption data (polypropylene panel in FIG 2 from^1^).

**Supplementary Methods: WB-PBPK models and the parameters used to derive the free interstitial concentrations in kidney:**

In pyelonephritis caused by *E. coli*, the bacteria reside in the extracellular (interstitium) area of the parenchyma. However, due to limited detailed information on the distribution of ciprofloxacin (CIP) and colistin (CST), substructures were lumped, e.g., parenchyma and tubules, in the simulation of concentrations.

The rate of change of CIP in kidney was computed as shown in equation S1 and S2,

| ${{dA}_{CIPkidney}}/{dt}=Q_{kidney}\times C_{p_{CIP}}-Q_{kidney}\times{A_{CIPkidney}}/{{V_{kidney}}/{K_{p_{CIPkidney}}}}-C_{p_{CIP}}\times{CL}_{R}$ | (S1) |
| --- | --- |
| ${CL}_{R}=\left( {CL}_{CR}\times60/1000 \right)\times f_{{u,p}_{CIP}}\times\left( 1+R_{SEC} \right)$ | (S2) |

where $A_{CIPkidney}$ is the amount of CIP in the kidney; $Q_{kidney}$ and $V_{kidney}$ are the blood flow and volume of kidney, respectively, and were set to be 73 L/h and 0.34 L representing a male with a body weight of 80 kg. $C_{p_{CIP}}$ is the CIP plasma concentration which was derived from a popPK model^2^. $K_{p_{CIPkidney}}$ is tissue (kidney)-to-plasma distribution coefficient for CIP and was set to the reported value of 8.09^3^. ${CL}_{R}$ is CIP renal clearance and relied on creatinine clearance (${CL}_{CR}$) which was here set to be 90 mL/min. CIP unbound fraction in plasma ($f_{{u,p}_{CIP}}$) was set to 0.65^3^ and the secretion factor into renal tubules ($R_{SEC}$) to 0.674^3^. To derive the unbound concentrations in the interstitium of kidney, total kidney concentration $\left( =A_{CIPkidney}/V_{kidney} \right)$ was multiplied by unbound fraction in tissue interstitium (*f*_u,t_) which was calculated according to equation S3^4^,

| $f_{u,t}=1/\left( 1+E/P\times\left( 1-f_{u,p} \right)/f_{u,p} \right)$ |  | (S3) |
| --- | --- | --- |

where E/P is the ratio of albumin in extracellular fluid to plasma and was set to 0.5 as reported for rat kidney^5^.

Similarly, yet further complex due to the transformation process from prodrug colistin methanesulfonate (CMS) to colistin (CST), equations for CST amount in kidney was calculated according to equations S4 and S5. Due to limited available information of some parameters of interest in humans, kidney tissue and tubular compartments were lumped assuming same concentration levels.

| ${{dA}_{CMSkidney}}/{dt}=Q_{kidney}\times C_{p_{CMS}}-Q_{kidney}\times{A_{CMSkidney}}/{{V_{kidney}}/{K_{p_{CMSkidney}}}}-{A_{CMSkidney}}/{K_{p_{CMSkidney}}\times k_{HYD}}-C_{p_{CMS}}\times{CL}_{R_{CMS}}$ | (S4) |
| --- | --- |
| ${{dA}_{CSTkidney}}/{dt}=Q_{kidney}\times C_{p_{CST}}-Q_{kidney}\times{A_{CSTkidney}}/{{V_{kidney}}/{K_{p_{CSTkidney}}}}+{A_{CMSkidney}}/{K_{p_{CMSkidney}}\times k_{HYD}}-{A_{CSTkidney}}/{K_{p_{CSTkidney}}\times k_{NR}}-C_{p_{CST}}\times{CL}_{R_{CST}}$ | (S5) |

where $A_{CMSkidney}$ and $A_{CSTkidney}$ are the amounts in the kidney of CMS and CST, respectively. $Q_{kidney}$ and $V_{kidney}$, as explained in the paragraph for CIP, shared the same values: 73 L/h and 0.34 L. $C_{p_{CMS}}$ and $C_{p_{CST}}$ are the CMS and CST plasma concentration derived from a popPK model^6^. $K_{p_{CMSkidney}}$ and $K_{p_{CSTkidney}}$ are kidney-to-plasma distribution coefficient for CMS and CST and the adopted values were 12.9 and 20.8^7^. CMS hydrolysis rate constant ($k_{HYD}$) and CST nonrenal intrinsic elimination rate constant ($k_{NR}$) were calculated according to equation S6 and S7, as earlier suggested given their relationship to tissue volume^8^.

| $k_{HYD}={{CL}_{NR_{CMS}}}/{WT}$  $k_{NR}={{CL}_{NR_{CST}}}/{WT}$ | (S6)  (S7) |
| --- | --- |

Where ${CL}_{NR_{CMS}}$ and ${CL}_{NR_{CST}}$ are nonrenal clearance of CMS (5.49 L/h) and CST (3.03 L/h) derived from a popPK model^6^. WT is the body weight of 80 kg and is the sum of the body tissue volume, assuming 1 kg = 1 L. ${CL}_{R_{CMS}}$ and ${CL}_{R_{CST}}$ are renal clearance of CMS (1.83 L/h) and CST (0.33 L/h), calculated from popPK^6^ using ${CL}_{CR}$ of 90 mL/min. Similarly as for CIP, the total CST kidney concentration $\left( A_{CSTkidney}/V_{kidney} \right)$ was multiplied by the unbound fraction in tissue (equation S3) with *f*_u,p_ equal to 0.34^6^. Considering CST distributes mainly extracellularly^9^, the derived unbound CST concentration in the kidney was further corrected by dividing interstitial volume fraction (IV_f_ = 0.196 for kidney^10^) to obtain the unbound CST concentrations in kidney interstitial fluid. This was not applied to CIP considering it accumulates in cells (i.e. intercellular) as well^11^. For CST, the popPK analysis utilized molar unit concentrations, where 1MU of administered CMS corresponded to 45.9 μmol. Subsequently, the colistin concentration was converted to mg/L, using the conversion factor of 1 mol/L equivalent to 1163 g/L.

**Reference:**

1. Karvanen, M., Malmberg, C., Lagerbäck, P., Friberg, L. E. & Cars, O. Colistin Is Extensively Lost during Standard In Vitro Experimental Conditions. *Antimicrob Agents Chemother* **61**, 1–9 (2017).

2. Khachman, D. *et al.* Optimizing ciprofloxacin dosing in intensive care unit patients through the use of population pharmacokinetic-pharmacodynamic analysis and monte carlo simulations. *Journal of Antimicrobial Chemotherapy* **66**, 1798–1809 (2011).

3. Sadiq, M. W. *et al.* A whole-body physiologically based pharmacokinetic (WB-PBPK) model of ciprofloxacin: a step towards predicting bacterial killing at sites of infection. *J Pharmacokinet Pharmacodyn* **44**, 69–79 (2017).

4. McNamara, P. J., Gibaldi, M. & Stoeckel, K. Fraction unbound in interstitial fluid. *J Pharm Sci* **72**, 834–6 (1983).

5. Björkman, S., Wada, D. R., Berling, B. M. & Benoni, G. Prediction of the disposition of midazolam in surgical patients by a physiologically based pharmacokinetic model. *J Pharm Sci* **90**, 1226–1241 (2001).

6. Kristoffersson, A. N. *et al.* Population pharmacokinetics of colistin and the relation to survival in critically ill patients infected with colistin susceptible and carbapenem-resistant bacteria. *Clinical Microbiology and Infection* **26**, 1644–1650 (2020).

7. Bouchene, S. Thesis: Physiologically Based Pharmacometric Models for Colistin and the Immune Response to Bacterial Infection. (2016).

8. Bouchene, S. *et al.* A Whole-Body Physiologically Based Pharmacokinetic Model for Colistin and Colistin Methanesulfonate in Rat. *Basic Clin Pharmacol Toxicol* **123**, 407–422 (2018).

9. Couet, W. *et al.* Pharmacokinetics of colistin and colistimethate sodium after a single 80-mg intravenous dose of CMS in young healthy volunteers. *Clin Pharmacol Ther* **89**, 875–9 (2011).

10. Bouchene, S. *et al.* A Whole-Body Physiologically Based Pharmacokinetic Model for Colistin and Colistin Methanesulfonate in Rat. *Basic Clin Pharmacol Toxicol* **123**, 407–422 (2018).

11. Mouton, J. W. *et al.* Tissue concentrations: Do we ever learn? *Journal of Antimicrobial Chemotherapy* **61**, 235–237 (2008).
